# Inspiratory muscle training for chronic critically ill patients: a systematic review and meta-analysis of randomized controlled trials

**DOI:** 10.31744/einstein_journal/2025RW1134

**Published:** 2025-07-02

**Authors:** Gustavo Rodrigues das Chagas, Aléxia Gabriela da Silva Vieira, Jamile Caroline Garbuglio de Araújo, Raquel Afonso Caserta Eid, Caroline Gomes Mól, Ricardo Kenji Nawa

**Affiliations:** 1 Hospital Israelita Albert Einstein São Paulo SP Brazil Hospital Israelita Albert Einstein, São Paulo, SP, Brazil.; 2 Faculdade Israelita de Ciências da Saúde Albert Einstein Hospital Israelita Albert Einstein São Paulo SP Brazil Faculdade Israelita de Ciências da Saúde Albert Einstein, Hospital Israelita Albert Einstein, São Paulo, SP, Brazil.

**Keywords:** Breathing exercises, Respiration, artificial, Tracheostomy, Critical illness, Chronic disease, Respiratory muscles, Muscle strength, Intubation, intratracheal

## Abstract

**Objective:**

To systematically review and analyze studies investigating the efficacy and safety of inspiratory muscle training in adult chronic critically ill patients.

**Methods:**

The MEDLINE, Embase, CENTRAL, LILACS, Clinical Trials Registry, and World Health Organization databases were queried on November 24, 2022 and January 22, 2025. The review was conducted in accordance with the PRISMA guidelines. RevMan V5.4 was used to analyze mean differences or standardized mean differences and 95% confidence intervals (95%CIs) for continuous variables and risk ratios with 95%CIs for dichotomous outcomes. The primary outcomes were inspiratory muscle strength, duration of mechanical ventilation, and severe adverse events; the secondary outcomes were hospital and intensive care unit mortality, intensive care unit and hospital lengths of stay, pulmonary function, non-serious adverse events, respiratory muscle mass, and functional status.

**Results:**

Seven studies (n=390 participants) were included in the analysis. There was a significant increase in inspiratory muscle strength with inspiratory muscle training *versus* usual care (mean difference, -8.37; 95%CI= -15.21 to -1.52), although the certainty of evidence was very low; when compared with sham interventions, there was no significant difference (mean difference, -4.26; 95%CI= -14.05 to 5.53), also with very low certainty of evidence. The results for pulmonary function, duration of mechanical ventilation, and mortality were imprecise, with very low certainty of evidence. The available evidence also indicates the potential safety benefit of inspiratory muscle training, although the certainty of evidence remains very low. Conclusion: We identified that inspiratory muscle training may improve inspiratory muscle strength, with little to no difference on duration of mechanical ventilation, pulmonary function and severe and non-serious adverse events, when compared to sham inspiratory muscle training and usual care. However, the certainty of the evidence is very low. Evidence regarding the impact of inspiratory muscle training on intensive care unit mortality and length of stay is uncertain. Prospero database registration ID CRD42022370750.

## INTRODUCTION

Recent advancements in healthcare and the integration of cutting-edge medical technologies have markedly enhanced the survival rates of critically ill patients admitted to intensive care units (ICUs) worldwide.^1,2^ However, this progress has also led to a growing population of critically ill patients requiring prolonged mechanical ventilation (MV) support and other high-complexity intensive care therapies.^1,3,4^

Chronic critically ill patients (CCIPs) are patients who experience an ICU stay of 8 days or more, coupled with conditions like prolonged MV, tracheostomy, severe infections, extensive wounds or multiple organ failure, ischemic stroke, intracerebral hemorrhage, or traumatic brain injury.^1-3,5^ Prolonged MV support for CCIPs is defined as the need for more than three consecutive weeks of support with a daily duration exceeding 6h.^3,6^ These clinical characteristics, combined with the prolonged MV weaning process, can affect both peripheral and respiratory muscles and contribute to the suboptimal clinical and functional outcomes associated with ICU-acquired weakness (ICUAW).^7-13^ This condition in turn is correlated with adverse prognostic outcomes characterized by poor mental health and quality of life in family members with high morbidity and mortality.^12,14^ Nonetheless, and even though ICUAW is becoming more common among ICU-admitted patients, its etiology and management are not well characterized.

Chronic critically ill patients also face significant challenges transitioning from the ICU to home settings, particularly in terms of functional recovery and impact on family members’ mental health and quality of life outcomes.^13,15^ Inspiratory muscle training (IMT) is recognized as an effective rehabilitation strategy to mitigate respiratory muscle loss and weakness in ICU patients and prevent ICUAW in respiratory muscles.^16-18^ Inspiratory muscle training specifically focuses on enhancing the strength and endurance of respiratory muscles, including the diaphragm and accessory muscles, and aims to alleviate symptoms such as dyspnea and improve the success rate of the weaning process.^19-21^ Although IMT has been demonstrated to be safe, feasible, and well tolerated in the general CCIP population, there is still considerable uncertainty in the literature regarding the prescription of IMT for these patients, mostly stemming from the wide variability in the load, frequency, and duration of IMT protocols, which can significantly affect outcomes.^20,22^

Thus, although numerous studies have demonstrated the efficacy of IMT for mechanically ventilated patients, its relevance for CCIPs remains unclear.

## OBJECTIVE

The purpose of this review was to evaluate the effectiveness of inspiratory muscle training for chronic critically ill patients and assess whether inspiratory muscle training is associated with enhancement of muscle strength in chronic critically ill patients.

## METHODS

This systematic review was conducted in accordance with the Preferred Reporting Items for Systematic Reviews and Meta-Analyses (PRISMA) guidelines,^23,24^ and followed the methodological recommendations of the Cochrane Collaboration Handbook.^25^

An initial search of the MEDLINE, Embase, CENTRAL, and LILACS databases was performed on November 24, 2022, with an updated search on January 22, 2025 (Tables 1S to 4S, Supplementary Material). Additionally, searches were also performed on the ClinicalTrials.gov registry website and the World Health Organization (WHO) International Clinical Trials Registry Platform to identify *‘ongoing’* and *‘unpublished’* studies (Tables 5S to 6S, Supplementary Material). There were no restrictions on language, date, or publication status. Only parallel randomized controlled trials (RCTs) were included; quasi-randomized trials were not included in this review.

The eligibility criteria were established using the Population, Intervention, Comparator, and Outcome (PICO) approach^26^ as follows: (P) population: adult patients meeting the criteria for CCIPs; (I) intervention: IMT, regardless of type, frequency, and duration; (C) comparator: general rehabilitation; usual or standard care; or no intervention; and (O) outcome: inspiratory muscle strength; MV duration; number of severe adverse events; ICU and hospital mortality; ICU and hospital length of stay; pulmonary function; non-serious adverse events; respiratory muscle mass/thickness; and functional status. Records from each individual study were collated so that each study was included only once.

Two investigators independently screened all titles and abstracts retrieved through the systematic search. A third investigator was consulted to resolve potential disagreements regarding the included articles if necessary. Thereafter, two investigators reviewed the articles for full-text assessment. Disagreements regarding eligibility were resolved through discussion.

The primary outcomes were inspiratory muscle strength, MV duration, and the number of severe adverse events. The secondary outcomes were ICU and hospital mortality, ICU and hospital length of stay, pulmonary function (*e.g*., total lung capacity and forced vital capacity), non-serious adverse events (*e.g*., respiratory muscle fatigue during or after the training as assessed based on clinical criteria such as increased respiratory rate, use of accessory respiratory muscles, and decrease in oxygen saturation), respiratory muscle mass/thickness (assessed based examinations such as point-of-care ultrasound assessment), and functional status (assessed based on criteria such as Perme ICU mobility score, ICU Mobility Scale score, Surgical ICU Optimal Mobilization Score, six-minute walking test result, thirty-second sit-to-stand test result).

Study characteristics and outcome data were independently extracted by two investigators and reviewed by a third investigator using a pre-defined data collection form. To characterize and assess the similarities of participants among included studies, we extracted and assessed details of experimental and control interventions, ranges of outcome measures, and assessment time points for each study.

The risk of bias of the outcomes was assessed using the Cochrane Risk of Bias 2 (RoB2) tool for randomized trials.^26,27^ Risk of bias was assessed in terms of five domains: (i) randomization process, (ii) deviations from intended interventions, (iii) missing outcome data, (iv) measurement of the outcome, and (v) selection of the reported result. For all included studies, a score indicating the level of risk of bias (‘low’, ‘some concerns’, or ‘high’) was assigned for each domain.

Mean differences (MDs) or standardized mean differences (SMDs) and 95% confidence intervals (95%CIs) were used to analyze continuous variables. For dichotomous outcomes, we calculated risk ratios (RRs) and 95%CIs. When possible, skewed data were adjusted for mean and standard deviation using Wan’s method and the Review Manager (RevMan) Calculator.^28^ When substantial heterogeneity was identified (*I*^2^ ≥ 50%), we conducted a pre-defined subgroup analysis for the number of IMT sessions.

RevMan version 5.4.1. (Copenhagen: The Nordic Cochrane Centre, The Cochrane Collaboration, 2020) was used for all analyses. The Grading of Recommendations Assessment, Development, and Evaluation (GRADE) system was used to assess and summarize the overall certainty of the current evidence for each outcome^29^ using the GRADEpro Guideline Development Tool.^30^

## RESULTS

A total of 3,531 records were identified in the initial search, and 2,890 unique records were screened after excluding duplicates. Following the assessment of titles, abstracts, and full-texts, 16 records originating from 7 distinct studies (n=390 participants)^31-37^ were included in the systematic review (Figure 1). The baseline characteristics of each included study are summarized in Table 1 and Tables 7S to 8S, Supplementary Material. The included studies were published between 2011 and 2022 and conducted in three countries: Brazil (n=5),^31,32,34,35,37^ the United States of America (n=1),^33^ and Belgium (n=1).^36^ The sample sizes ranged from 10 to 101 participants. Three studies^31,33,34^ utilized the Threshold^®^ Inspiratory Muscle Trainer device and four studies^32,35-37^ employed the POWERbreathe^®^ device. The initial training load intensity was between 20 and 40% of maximum inspiratory pressure (MIP).

**Figure 1:**
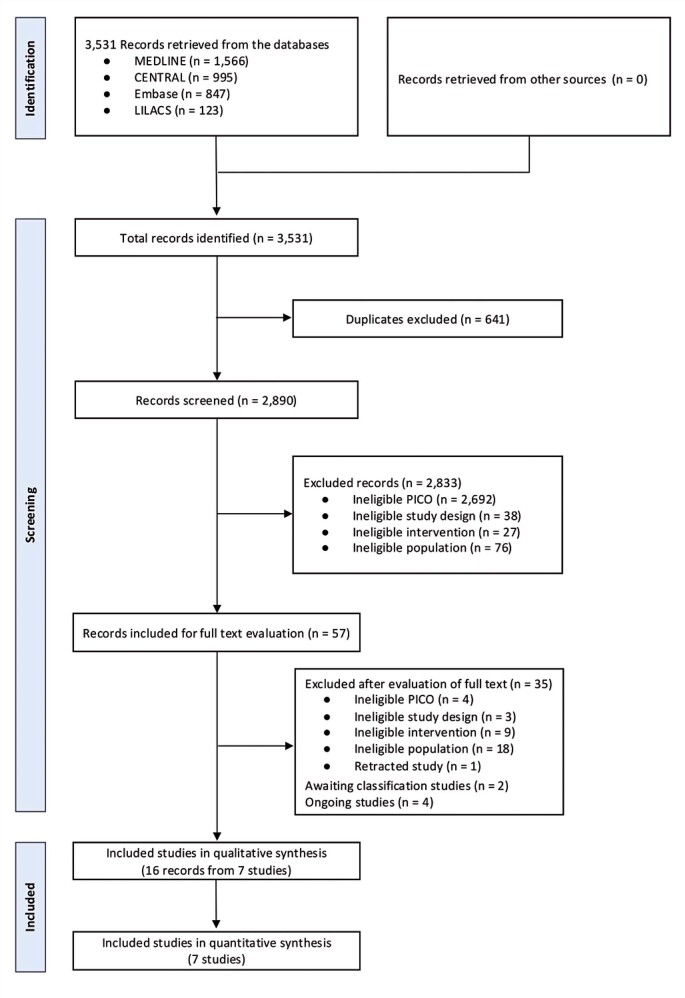
PRISMA flow chart of studies selection

CENTRAL: Cochrane Central Register of Controlled Trials; EMBASE: Excerpta Medica dataBASE; MEDLINE: Medical Literature Analysis and Retrieval System Online; PICO: Patient Intervention Comparator Outcome.

**Table 1:** Summary of included randomized controlled trials

Author (year) Country	Number of participants	Participant details	Intervention and severity score	
			Experimental Group	Control Group
Condessa et al. (2013)^(31)^ Brazil	77	- Age (y), mean (SD) = Experimental Group: 64 (17), Control Group: 65 (15) - Gender, (male), n (%) = Experimental Group: 23 (51), Control Group: 28 (60) - Eligibility = age ≥18 years; MV >48 h; ready for weaning; PEEP 5-7 cmH_2_O; hemodynamically stable without use of vasopressors or sedatives	- IMT + usual care - APACHE II score, mean (SD) = 23 (8)	- Usual care - APACHE II score, mean (SD) = 23 (8)
da Silva Guimarães et al. (2021)^(32)^ Brazil	101	- Age (y), mean (SD) = Experimental Group: 63 (16), Control Group: 69 (16) - Gender, (M/F), n (%) = Experimental Group: 24 (50) / 24 (50), Control Group: 25 (47) / 28 (53) - Eligibility = age 18-86 years; TCT; prolonged weaning; cough reflex; no excessive secretion; no infection; stable cardiovascular; no or minimal use of vasopressors; Hb >7-10 g/dL; SaO_2_ > 90% with an FiO_2_ ≤40% or P/F ≥150 with PEEP ≤ 5-8 cm H_2_O; RR ≤ 35 breaths/min; PSV ≤ 20 cmH_2_O; pH > 7.30; T <38 °C; consciousness level: alert or non-alert	- IMT + Spontaneous breathing with ‘T-piece’ - APACHE II score, median [IQR] = 29 [26–32]	- Spontaneous breathing with ‘T-piece’ - APACHE II score, median [IQR] = 27 [22–31]
Martin et al. (2011)^(33)^ United States	69	- Age (y), mean (SD) = Experimental Group: 65.6 (11.7), Control Group: 65.1 (10.7) - Gender (M/F), n = Experimental Group: 16/19, Control Group: 15/19 - Eligibility = age ≥18 years; BMI <40 kg/m^2^; T ≤38.5 °C; PaO_2_ >60mmHg with FiO_2_ ≤50%; ready for weaning; hemodynamically stable; able to follow commands; A/C, SIMV or PSV mode; TCT, SIMV ≤6 breaths/min, PSV ≤15cmH_2_O and PEEP ≤10cmH_2_O; unable to sustain unsupported breathing for at least 72 h consecutively	- IMT - SAPS II at study start, mean (SD) = 33.5 (8.6)	- Sham group - SAPS II at study start, mean (SD) = 33 (8.6)
Pascotini et al. (2014)^(34)^ Brazil	14	- Age (y), mean (SD) = Experimental Group: 67 (13.9), Control Group: 72.4 (11.9) - Gender, (M/F), n = Experimental Group: 0/7, Control Group: 3/4 - Eligibility = age ≥ 40 years; TCT cannula; ready for weaning from MV support	- IMT + usual care - ICU scoring system = NR	- Usual care - ICU scoring system = NR
Melo et al. (2017)^(35)^ Brazil	10	- Age (y), mean (SD) = 35 (14) - Gender, (male), (%) = 75% - Eligibility = MV ≥ 7 days	- IMT + usual care - APACHE II, median [IQR] = 17 [14-22]	- Usual care (EM protocol + respiratory therapy) - APACHE II, median [IQR] = 17 [14-22]
Van Hollebeke et al. (2022)^(36)^ Belgium	41	- Age (y), mean (SD) = Experimental Group: 52 (18), Control Group: 64 (7) - Gender, (M/F), n = Experimental Group: 13/9, Control Group = 9/10 - Eligibility = unsuccessful wean from MV within 24 h after the first separation attempt; met all ‘readiness to wean’ criteria; unable to be weaned within 24 h after the first failed separation attempt; able to follow commands to perform the IMT	- IMT (high-intensity) APACHE II, mean (SD) = 19 (8)	- Sham (low-intensity IMT) - APACHE II, mean (SD) = 20 (6)
Roceto Ratti et al. (2022)^(37)^ Brazil	78	- Age (y) = Experimental Group 1: 52 (17.3), Experimental Group 2: 57 (15.57), Control Group: 56 (18.29) - Gender, (M/F), % = Experimental Group 1: 59/40, Experimental Group 2: 70/29, Control Group: 81/18 - Eligibility = without continuous sedation or analgesic; PaO_2_ >60 mmHg with FiO_2_ 60%; OI ≥ 100; TCT cannula; A/C, SIMV or PSV mode; PEEP ≤10cmH_2_O; RR ≤ 30 breaths/min; SpO_2_ ≥90%; clinically stable for weaning; hemodynamically stable within the 24 h previous to the IMT; MBP 80-100 mmHg; HR 60-120 bpm; T 36.5-38.5ºC; absence of degenerative or any other neuromuscular disease	- Experimental Group 1 = IMT (automatic) - APACHE II, mean (SD) = 14 (6.53) - Experimental Group 2 = IMT (manual) - APACHE II, mean (SD) = 18 (8.04)	- Spontaneous breathing with ‘T-piece’ APACHE II, mean (SD) = 17 (5.31)

A/C: assist control; APACHE: Acute Physiology and Chronic Health Evaluation; BMI: body mass index; bpm: beats per minute; FiO_2_: fraction of inspired oxygen; Hb: hemoglobin; HR: heart rate; ICU: intensive care unit; IMT: inspiratory muscle training; MBP: mean blood pressure; MIP: maximum inspiratory pressure; MV: mechanical ventilation; OI: oxygenation index; P/F: ratio of the PaO_2_ in arterial blood by the fraction of inspired oxygen; PEEP: positive end-expiratory pressure; post-op = postoperative; PSV: pressure support ventilation; RR: respiratory rate; RSBI: Rapid Shallow Breathing Index; SaO_2_: arterial oxygen saturation; SAPS: simplified acute physiology score; SD: standard deviation; SIMV: synchronized intermittent mandatory ventilation; T: temperature; TCT: tracheostomy; Vt: tidal volume; y: years.

Four studies^31,33,36,37^ described details of the randomization process, deviations from intended interventions, and missing outcome data. However, three studies^32,34,35)^ were judged as having a ‘high risk’ of bias for the inspiratory muscle strength outcome due to insufficient information on the randomization process and lack of information regarding assessor blinding. Three studies^31,34,35^ were judged as having a ‘some concerns’ level risk of bias for the following outcomes: MV duration, number of severe adverse events, ICU mortality, ICU length of stay, and non-serious adverse events. The overall risk of bias in the included studies is summarized in Figure 1S, Supplementary Material.

Four studies^31,32,34,37^ investigating inspiratory muscle strength were included in the quantitative analysis (Figure 2). The IMT group had a higher increase in the inspiratory muscle strength than usual care group (MD, -8.37; 95%CI= -15.21 to -1.52), with very low certainty of evidence. The certainty of evidence was double-downgraded due to serious imprecision, a small number of participants, and moderate inconsistency (*I*^2^ = 39%) (Table 9S, Supplementary Material). Moderate heterogeneity was found and explored through subgroup analyses focusing on the number of repetitions per session (Figure 2S, Supplementary Material). The effect was not significantly different when comparing the IMT and usual care groups, and low heterogeneity (*I*^2^ = 0%) was noted. A significant increase in inspiratory muscle strength was observed for participants performing more than 50 repetitions per IMT session (MD, -10.88; 95%CI= -22.03 to 0.26), although considerable heterogeneity (*I*^2^ = 75%) was noted.

**Figure 2:**
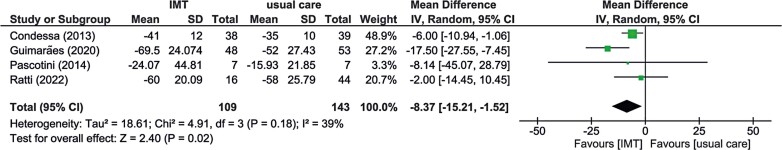
Forest plot of inspiratory muscle strength: inspiratory muscle training versus usual care

95%CI: 95%confidence interval; IMT: inspiratory muscle training; SD: standard deviation.

**Figure 3:**
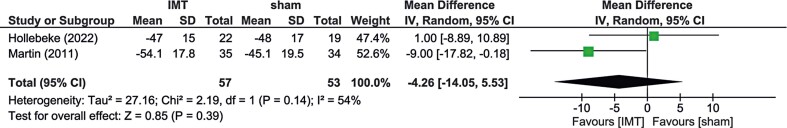
Forest plot of inspiratory muscle strength: inspiratory muscle training versus sham

Two studies (33,36) compared IMT with sham interventions and did not find significant differences in MIP (MD, -4.26; 95%CI= -14.05 to 5.53) with moderate heterogeneity (*I*^2^ = 54%) and very low certainty of evidence (Figure 3). The certainty of evidence was downgraded due to imprecision, few participants, and inconsistency (Table 9S, Supplementary Material). In one study,^33^ a short intervention period characterized by a higher number of repetitions per session was used, with a favorable trend to the IMT group (Figure 3). In contrast, another study^36^ reported the longest duration of intervention, ranging up to 28 days or successful weaning from MV, and the results showed a large confidence interval (Figure 3).

95% CI: 95% confidence interval; IMT: inspiratory muscle training; SD: standard deviation.

Three studies^31,34,37^ investigated the duration of MV. Quantitative analysis was not performed because of data heterogeneity, especially regarding the unit of measure (days or hours) and the final cutoff point used to determine the total duration of MV. The certainty of evidence was classified as very low and double-downgraded because of very serious imprecision (Table 9S, Supplemantary Material). One study^37^ (n=104 participants) investigated two interventions (automatic and manual IMT) in addition to the control treatment and reported a decrease in MV duration (days) (median [IQR]: usual care, 24.5 [15.75-32.25]; automatic IMT, 18 [15.25-26.50]; manual IMT, 14.5 [12-21.75];^37^ the authors measured MV duration from the time of tracheostomy until continuous spontaneous breathing for 48h.^37^ Two other studies reported a slight difference^31^ and no difference^34^ in the duration of MV when comparing IMT with usual care.

Two studies^31,33^ assessed the safety of IMT, although both used a different comparator (usual care^31^ and sham).^33^ The details of recording and registering adverse events were reported in only one study,^31^ which focused on adverse events related to hemodynamic changes. None of the studies reported any evidence of adverse events related to the use of IMT for CCIPs. Despite this positive trend, the sample size was small, and the results should therefore be interpreted with caution. The certainty of the evidence was very low, and we doubled downgraded it because of very serious imprecision (Table 9S, Supplementary Material).

Four studies^31-33,37^ evaluated mortality with different follow-up periods ranging from 7 days to 1 year. Two studies^35,37^ reported length of stay, one study^35^ did not find any differences between the IMT and Control Groups whereas the other^38^ showed a small difference between the automatic IMT and Control Groups. However, owing to the short period of the IMT intervention compared to the total duration of hospital care, these results may not reflect the actual effects of the intervention on mortality and length of stay, and thus, a quantitative analysis was not performed for these outcomes. Only one study^36^ evaluated pulmonary function, based on forced vital capacity, and reported improvement in the IMT group when compared to the Control Group, although the certainty of evidence was uncertain due to imprecision of the results (Table 9S, Suplemmentary Material). Some outcomes specified in the registered protocol, specifically hospital mortality, length of hospital stay, respiratory muscle mass/thickness, and functional status, were not assessed in any of the studies included in this review.

## DISCUSSION

To the best of our knowledge, this is the first study to review the effects of IMT on CCIPs. This systematic review and meta-analysis showed that IMT may enhance inspiratory muscle strength, presenting preliminary results of reduced MV duration and improved pulmonary function, with no reports of severe or non-serious adverse events. However, evidence regarding the impact of IMT on ICU mortality and length of stay is uncertain owing to the imprecision of and short duration of interventions in the included studies.

Inspiratory muscle weakness is a common complication among CCIPs. It is considered a risk factor for a prolonged MV weaning process and may increase the incidence of ventilator-associated pneumonia, predisposing patients to poor clinical and physical outcomes.^2,39,40^ The results of this systematic review showed that IMT slightly increased inspiratory muscle strength in CCIPs when compared to usual care patients, with no significant difference between the IMT and sham groups. A previous review also reported a moderate yet potentially impactful enhancement of inspiratory muscle strength,^22^ but there was high heterogeneity among the included studies due to considerable variability between IMT protocols with regard to dose, intensity, and frequency.^19,41,42^ The present review also found improvements in inspiratory muscle strength in patients who performed more repetitions per session. Most of the studies included in this review employed inspiratory threshold loading to address IMT protocols. The literature also supports the hypothesis that IMT can improve inspiratory muscle strength, exercise performance capacity, and quality of life in patients with chronic conditions such as chronic obstructive pulmonary disease,^43^ heart failure,^44^ asthma,^45^ and cystic fibrosis.^46^ Additionally, these benefits have been reported in patients with spinal cord injury,^47^ multiple sclerosis,^48^ and neuromuscular diseases.^49^ For patients undergoing elective open cardiac surgery, IMT improved inspiratory muscle strength and reduced the risk of postoperative pulmonary complications and the length of hospital stay.^50^

A reduction in MV duration has also been reported in a previous systematic review that included patients who underwent IMT sessions while being admitted to the ICU.^22^ Although the treatment effect did not persist after excluding studies with a ‘serious risk of bias’, IMT was associated with a reduction in duration of weaning from MV support.^22^ Our findings also corroborate those of a previous review that reported no clear evidence regarding the effects of IMT on MV duration. Additionally, none of the studies included in this review reported the occurrence of adverse events (mild, moderate, or severe), and previous literature corroborates these findings, as they also report a low incidence of adverse events in patients who underwent IMT. Although infrequent, the commonly reported complications of IMT sessions in these studies are bradycardia, syncope, paradoxical breathing, tachypnea, desaturation, and hemodynamic instability.^22^ The feasibility and tolerance of IMT applications have been demonstrated;^19,21,22^ however, the evidence remains uncertain for CCIPs.

This systematic review and meta-analysis provided important insights and contributions to literature by presenting evidence regarding the effectiveness of IMT for CCIPs. The strengths of this review include clearly defined search criteria and methodology following guidelines regarding best practices for systematic review. By ensuring high methodological rigor—employing a sensitive and broad search strategy and independent selection, extraction, and analysis of data by two investigators), we considerably reduced the chances of excluding any relevant study. Additionally, information on ‘ongoing studies’ is also presented to help provide readers a more complete overview of this topic.

However, this review has some limitations. First, the certainty of evidence was judged as ‘very low’ due to imprecision and ‘moderate’ to ‘high’ risk of bias for most of the outcomes assessed. The overall certainty of the evidence in the included studies was compromised because of the absence of transparency and missing information on the randomization methods used. Second, the short intervention and follow-up periods and heterogeneity among IMT protocols may have affected the findings and limited the possibility of performing a meta-analysis. Third, in-hospital mortality and hospital stay, muscle mass, and functional status were not assessed as outcomes in any of the included studies, although these were planned for in the systematic review protocol. Fourth, due to the small number of included studies, the findings of this review should be interpreted with caution. Therefore, future RCTs should focus on extending the duration of IMT interventions and determining the effects of IMT on mortality, length of hospital stay, functional status, and respiratory muscle mass.

In summary, our findings suggest that IMT for CCIPs is associated with increased inspiratory muscle strength when the number of repetitions is appropriately high; furthermore, IMT is not associated with adverse events and may reduce MV duration. However, the certainty of the evidence remains low, and future studies are needed to investigate the benefits of respiratory muscle training for CCIPs, focusing on the dose, duration, and intensity of training.

## Figures and Tables

**Figure 1S:**
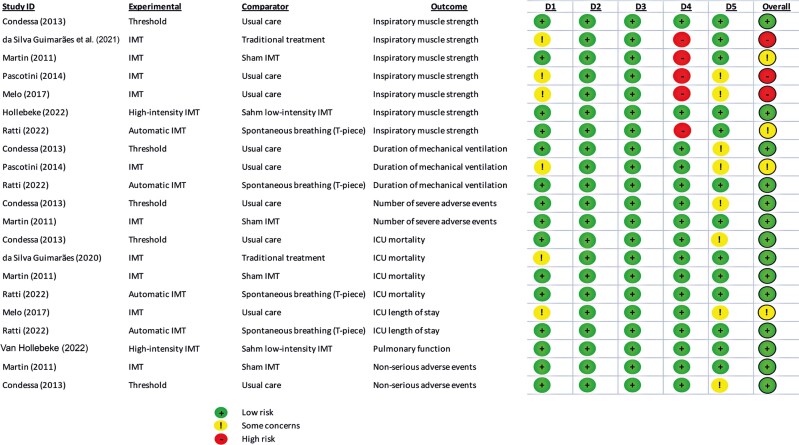
Risk of bias in the included Risk of bias in the included randomized controlled trials

D1: randomization process; D2: deviations from the intended interventions; D3: missing outcome data; D4: measurement of the outcome; D5: selection of the reported result; ICU: intensive care unit; IMT: inspiratory muscle training.

**Figure 2S:**
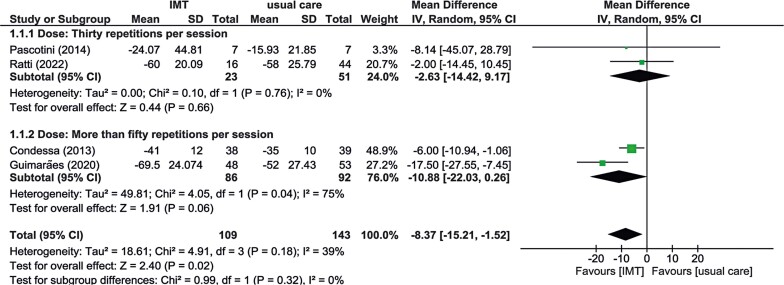
Forest plot of subgroup analysis of inspiratory muscle strength based on the number of repetitions per session

95%CI: 95% confidence interval; IMT: inspiratory muscle training; SD: standard deviation.

## SUPPLEMENTARY MATERIAL

**Inspiratory muscle training for chronic critically ill patients: a systematic review and meta-analysis of randomized controlled trials**
Gustavo Rodrigues das Chagas, Aléxia Gabriela da Silva Vieira, Jamile Caroline Garbuglio de Araújo, Raquel Afonso Caserta Eid, Caroline Gomes Mól, Ricardo Kenji Nawa
**DOI: 10.31744/einstein_journal/2025RW1134**
**Table 1S:** Detailed search strategy for the MEDLINE database

Number	Search strategy
	**Search terms**
#1	“Respiration, Artificial”[Mesh] OR “Tracheostomy”[Mesh] OR “Intubation, Intratracheal”[Mesh] OR Artificial Respiration*[tiab] OR Mechanical Ventilation*[tiab] OR artificial ventilat*[tiab] OR intubat*[tiab] OR Tracheostom*[tiab]
#2	“Breathing Exercises”[Mesh] OR Inspirat* exercis*[tiab] OR Inspirat* train*[tiab] OR Inspirat* musc*[tiab] OR respirat* exercis*[tiab] OR respirat* musc*[tiab] OR respirat* train*[tiab] OR ventilat* exercis*[tiab] OR ventilat* musc*[tiab] OR ventilat* train*[tiab] OR breath* exercis*[tiab] OR breath* musc*[tiab] OR breath* train*[tiab] OR IMT[tiab] OR RMT[tiab] OR threshold[tiab] OR resist* load*[tiab] OR resist* device*[tiab] OR powerbreath*[tiab]
#3	((clinical[Title/Abstract] AND trial[Title/Abstract]) OR clinical trials as topic[MeSH Terms] OR clinical trial[Publication Type] OR random*[Title/Abstract] OR random allocation[MeSH Terms] OR therapeutic use[MeSH Subheading])
#4	#1 AND #2 AND #3

MEDLINE: Medical Literature Analysis and Retrieval System Online. **Table 2S:** Detailed search strategy for the EMBASE database

Number	Search strategy
	**Search terms**
#1	‘artificial ventilation’/exp OR ‘tracheostomy’/exp OR ‘endotracheal intubation’/exp OR ‘artificial respiration*’:ab,ti OR ‘mechanical ventilation*’:ab,ti OR ‘artificial ventilat*’:ab,ti OR intubat*:ab,ti OR tracheostom*:ab,ti
#2	‘breathing exercise’/exp OR ‘inspirat* exercis*’:ab,ti OR ‘inspirat* train*’:ab,ti OR ‘inspirat* musc*’:ab,ti OR ‘respirat* exercis*’:ab,ti OR ‘respirat* musc*’:ab,ti OR ‘respirat* train*’:ab,ti OR ‘ventilat* exercis*’:ab,ti OR ‘ventilat* musc*’:ab,ti OR ‘ventilat* train*’:ab,ti OR ‘breath* exercis*’:ab,ti OR ‘breath* musc*’:ab,ti OR ‘breath* train*’:ab,ti OR imt:ab,ti OR rmt:ab,ti OR threshold:ab,ti OR ‘resist* load*’:ab,ti OR ‘resist* device*’:ab,ti OR powerbreath*:ab,ti
#3	‘crossover procedure’:de OR ‘double-blind procedure’:de OR ‘randomized controlled trial’:de OR ‘single-blind procedure’:de OR (random* OR factorial* OR crossover* OR cross NEXT/1 over* OR placebo* OR doubl* NEAR/1 blind* OR singl* NEAR/1 blind* OR assign* OR allocat* OR volunteer*):de,ab,ti
#4	#1 AND #2 AND #3
#5	#4 AND [embase]/lim NOT ([embase]/lim AND [medline]/lim)

EMBASE: Excerpta Medica dataBASE. **Table 3S:** Detailed search strategy for the CENTRAL database

Number	Search strategy
	**Search terms**
#1	[mh “Respiration, Artificial”] OR [mh Tracheostomy] OR [mh “Intubation, Intratracheal”] OR (Artificial NEXT Respiration* OR Mechanical NEXT Ventilation* OR artificial NEXT ventilat* OR intubat* OR Tracheostom*):ti,ab
#2	[mh “Breathing Exercises”] OR (Inspirat* NEXT exercis* OR Inspirat* NEXT train* OR Inspirat* NEXT musc* OR respirat* NEXT exercis* OR respirat* NEXT musc* OR respirat* NEXT train* OR ventilat* NEXT exercis* OR ventilat* NEXT musc* OR ventilat* NEXT train* OR breath* NEXT exercis* OR breath* NEXT musc* OR breath* NEXT train* OR IMT OR RMT OR threshold OR resist* NEXT load* OR resist* NEXT device* OR powerbreath*):ti,ab
#3	#1 AND #2

CENTRAL: Cochrane Central Register of Controlled Trials. **Table 4S:** Detailed search strategy for the LILACS database

Number	Search strategy
	**Search terms**
#1	MH:”Respiração Artificial” OR MH:”Respiration, Artificial” OR MH:”Respiración Artificial” OR MH:E02.041.625$ OR MH:E02.365.647.729$ OR MH:E02.880.820$ OR MH:”Traqueostomia” OR MH:”Tracheostomy” OR MH:”Traqueostomía” OR MH:E02.041.750$ OR MH:E04.579.935$ OR MH:E04.580.900$ OR MH:E04.928.780$ OR MH:”Intubação Intratraqueal” OR MH:”Intubation, Intratracheal” OR MH:”Intubación Intratraqueal” OR MH:E02.041.500$ OR MH:E02.585.578$ OR MH:E05.497.578$ OR (TW:Artificial Respiration*) OR (TW:Mechanical Ventilation*) OR (TW:artificial ventilat*) OR TW:intubat* OR TW:Tracheostom*
#2	MH:”Exercícios Respiratórios” OR MH:”Breathing Exercises” OR MH:”Ejercicios Respiratorios” OR MH:E02.190.525.186$ OR MH:E02.779.474.124$ OR (TW:Inspirat* exercis*) OR (TW:Inspirat* train*) OR (TW:Inspirat* musc*) OR (TW:respirat* exercis*) OR (TW:respirat* musc*) OR (TW:respirat* train*) OR (TW:ventilat* exercis*) OR (TW:ventilat* musc*) OR (TW:ventilat* train*) OR (TW:breath* exercis*) OR (TW:breath* musc*) OR (TW:breath* train*) OR TW:IMT OR TW:RMT OR TW:threshold OR (TW:resist* load*) OR (TW:resist* device*) OR (TW:powerbreath*)
#3	(mh:(“Randomized Controlled Trials as Topic” OR “Controlled Clinical Trials as Topic” OR “Random Allocation” OR “Double-Blind Method” OR “Single-Blind Method” OR “Placebos” OR “Multicenter Studies as Topic” OR “Cross-Over Studies” OR “Pragmatic Clinical Trials as Topic”) OR pt:(“Randomized Controlled Trial” OR “Controlled Clinical Trial” OR “Multicenter Studies” OR “Pragmatic Clinical Trial”) OR ti:(random* OR aleatori* OR placebo*) OR (ti:(“clinical trial” OR “ensayo clinico” OR “ensaio clinico”) AND tw:(control* OR random* OR aleatori* OR placebo*)) OR (ti:(“cross-Over” OR multicenter OR multicentric*) AND ti:(study OR studies OR estud*)) OR ab:(randomi* OR aleatori* OR placebo*) OR (ab:(“clinical trial” OR “ensayo clinico” OR “ensaio clinico”) AND tw:(control* OR random* OR aleatori* OR placebo*)) OR (ab:(“cross-Over” OR multicenter OR multicentric*) AND ab:(study OR studies OR estud*)) OR (tw:(simple* OR singl* OR duplo* OR doble* OR doubl* OR trebl* OR tripl*) AND tw:(cego OR ciego OR blind OR mask OR dumm*))) AND NOT ((mh:”animals” AND NOT mh:”humans”) OR mh:”Retrospective Studies”)
#4	#1 AND #2 AND #3
#5	( db:(“LILACS”))

LILACS: Latin American and Caribbean Literature on Health Sciences Database.

*Filtro da base de dados BVS: ( db:(“LILACS”). **Table 5S:** Details of ongoing studies

Principal investigator (Study initiation year)	Trial registry ID	Country	Last update	Recruitment status	Methods
Langer et al. (2020)^(1)^	NCT04658498	Belgium	June 6, 2024	Recruiting	- Experimental 1: usual care + High-intensity IMT - Experimental 2: usual care + Low-intensity IMT (sham IMT) - Control: usual care
Borraz et al. (2020)^(2)^	NCT04347317	Spain	April 19, 2021	Not yet recruiting	- Experimental: High intensity IMT - Control: Low intensity IMT
Carvalho et al. (2019)^(3)^	NCT03758573	Brazil	May 10, 2023	Recruiting	- Experimental: IMT - Control: Intensive Physiotherapy
Kothapalli et al. (2019)^(4)^	ISRCTN15425727	Ireland	October 31, 2022	Completed	- Experimental 1: 2 weeks of IMT - Experimental 2: 2 weeks of both expiratory and IMT
Langer et al. (2017)^(5)^	NCT03240263	Belgium	June 06, 2024	Completed	- Experimental: IMT (high IMT) - Control: Sham (IMT low intensity)
Morris et al. (2013)^(6)^	NCT02003053	United States	September 07, 2018	Completed	- Experimental: IMT (start with 30% of MIP, 5 min. 2x/day with increments of 10 % every day for 7 days/week until liberation from MV or D/C - Control: Sham IMT (sham device 5 min. 2x/day for 7 days/week until liberation from MV or D/C

IMT: inspiratory muscle training; ISRCTN: International Standard Randomised Controlled Trial Number; min: minutes; MV: mechanical ventilation; NCT: National Clinical Trials. REFERENCES 1. ClinicalTrials.gov. Identifier: NCT04658498. Improving Our Understanding of Respiratory Muscle Training to Facilitate Weaning From Mechanical Ventilation in the ICU (TrainToWean). Bethesda (MD): National Library of Medicine (US); 2024 [cited 2024 Nov 10]. Available from: https://clinicaltrials.gov/study/NCT04658498 2. ClinicalTrials.gov. Identifier: NCT04347317. Can High Intensity Inspiratory Muscle Training Improve Inspiratory Muscle Strength and Accelerate Weaning in Medical Patients With Difficulty on Weaning?. Bethesda (MD): National Library of Medicine (US); 2021 [cited 2024 Nov 10]. Available from: https://clinicaltrials.gov/study/NCT04347317?term=NCT04347317&rank=1 3. ClinicalTrials.gov. Identifier: NCT03758573. Effectiveness Inspirational Muscle Training (IMTversusMV). Bethesda (MD): National Library of Medicine (US); 2023 [cited 2024 Nov 10]. Available from: https://clinicaltrials.gov/study/NCT03758573?term=NCT03758573&rank=1 4. ISRCTN Registry. ISRCTN15425727. Comparison of the effectiveness of two different respiratory exercise training methods in prolonged ventilated patients. Ireland: ISRCTN Registry; 2021 [cited 2024 Nov 10]. Available from: https://www.isrctn.com/ISRCTN15425727 5. ClinicalTrials.gov. Identifier: NCT03240363. Inspiratory Muscle Training in Difficult to Wean Patients. Bethesda (MD): National Library of Medicine (US); 2024 [cited 2024 Nov 10]. Available from: https://clinicaltrials.gov/study/NC T03240263?term=NCT03240263&rank=1 6. ClinicalTrials.gov. Identifier: NCT0 2003053. A Randomized, Controlled Trial of Inspiratory Muscle Training (IMT)in the ICU and CCU. Bethesda (MD): National Library of Medicine (US); 2018 [cited 2024 Nov 10]. Available from: https://clinicaltrials.gov/study/NCT02003053?term=NCT02003053&rank=1 **Table 6S:** Awaiting classification studies

Author (Study initiation year) [Country]	Participants	Interventions	Outcomes	Notes
Shosholcheva et al. (2016)^(1)^ [Macedonia]	- n = 34 - Experimental, n = 19 - Control, n = 15	- Experimental: Physical rehabilitation in the first 12-24 h - Control: Later rehabilitation	- Days of MV - Evaluation of changes in APACHE II score - Days to discharge from ICU	- Trial published in the Meeting abstracts. - The intervention information provided is insufficient to establish what kind of IMT was adopted in the study
Shrestha et al. (2014)^(2)^ [USA]	- n = 7 - Experimental, n = 4 - Control, n = 3	- Experimental: IMT via ETT - Control: Sham	- MIP - Reintubation rate - Vital signs monitoring	- It is not clear how long the included patients were on MV before screening for SBT - We did not have access to the full text

APACHE: Acute Physiology and Chronic Health Evaluation; ETT: endotracheal tube; ICU: intensive care unit; IMT: inspiratory muscle training; MIP: maximum inspiratory pressure; MV: mechanical ventilation; SBT: spontaneous breathing trial. REFERENCES 1. Condessa RL, Brauner JS, Saul AL, Baptista M, Silva AC, Vieira SR. Inspiratory muscle training did not accelerate weaning from mechanical ventilation but did improve tidal volume and maximal respiratory pressures: a randomised trial. J Physiother. 2013;59(2):101-7. 2. Roceto Ratti LD, Marques Tonella R, Castilho de Figueir do L, Bredda Saad IA, Eiras Falcão AL, Martins de Oliveira PP. Inspiratory Muscle Training Strategies in Tracheostomized Critically Ill Individuals. Respir Care. 2022;67(8):939-48. **Table 7S:** Summary of admission causes and outcomes

Author (year) Country	Causes of ICU admission	Outcomes
Condessa et al. (2013)^(1)^ Brazil	- Cause for ICU admission, n (%) Experimental Group COPD: 22 (49); trauma: 1 (2); immunosuppression: 9 (20); post-op: 4 (9); pneumonia: 9 (20) Control Group COPD: 18 (38), immunosuppression: 10 (21), post-op: 7 (15), pneumonia: 12 (26).	- Duration of weaning from MV - Inspiratory and expiratory muscle strength - Vt - RSBI
da Silva Guimarães et al. (2021)^(2)^ Brazil	- Cause for ICU admission, n (%) Experimental Group sepsis: 25 (52.1), pneumonia: 11 (22.9), COPD: 4 (8.3), stroke 3 (6.2), ARDS: 2 (4.2), brain trauma: 1 (2.1), CPR: 2 (4.2) Control Group sepsis: 26 (49.1); pneumonia: 11 (20.8); COPD: 4 (7.5); stroke: 5 (9.4); ARDS: 2 (3.8), brain trauma: 2 (3.8), CPR: 3 (5.6)	- Successful weaning - ICU survival rate (death was computed irrespective of the situation of the participant regarding MV dependence) - Duration of weaning from MV in days (counted from the start of the MV weaning until complete liberation from the ventilator) - Changes in the TIE index - MIP
Martin et al. (2011)^(3)^ United States	- Cause for ICU admission, n Experimental Group ARDS (3); AAA repair (2); esophageal surgery (6); GI surgery (6); hepatobiliary surgery (4); liver transplantation (2); acute congestive HR, MI or unstable angina, interstitial disease, acute intracranial hemorrhage, pancreatitis, sepsis with shock, dissecting/ruptured aorta, peripheral artery bypass graft, other cardiovascular surgery, esophageal not neo surgery, hepatobiliary surgery, full-thickness burns/skin grafting (1 each) Control Group sepsis (2); AAA repair (2); multiple simultaneous procedures (2); esophageal surgery: 3; gastrointestinal surgery (7); craniotomy (4); spinal surgery (2); orthopedic surgery (2); pneumothorax, pulmonary vasculitis, pancreatitis, dissecting/ruptured aorta, cardiac valve replacement, esophageal surgery, GI surgery, hepatobiliary surgery, spinal cord injury, multiple simultaneous procedures, liver transplantation, full-thickness burns/skin grafting (1 each)	- MIP - Weaning rate - MV support prior to starting intervention - Adverse events
Pascotini et al. (2014)^(4)^ Brazil	- Cause for ICU admission, n Experimental Group TBI (3); CVA (4) Control Group TBI (2); CVA (5)	- MIP - MEP - Vt - RR - HR
Melo et al. (2017)^(5)^ Brazil	- Cause for ICU admission, (%) Polytrauma (58)	- MIP - ICU LOS - Duration of weaning from MV
Van Hollebeke et al. (2022)^(6)^ Belgium	- Cause for ICU admission Experimental Group Transplantation (14); pneumonia (4); HF (2); hematologic (1); maxillofacial surgery (1) Control Group Transplantation (9); lung surgery (1); pneumonia (2); heart failure (3); esophageal surgery (1); polytrauma (1), organophosphate intoxication (1)	- MIP - Inspiratory muscle oxygenation parameters Total (Hb + Mb)
Roceto Ratti et al. (2022)^(7)^ Brazil	- Cause for ICU admission, (%) Experimental Group 1 Reduced consciousness (50); ARF (27.3); post-op (18.2); hemodynamic instability (4.5) Experimental Group 2 Reduced consciousness (37.5); ARF (58.3); hemodynamic instability (4.2) Control Group Reduced consciousness (47); ARF (28); post-op (20.7); CRA (3.8)	- ICU LOS - Duration of weaning from MV - Time from TCT to achievement of continuous spontaneous breathing for 48 h - MIP - RSBI AAA: abdominal aortic aneurysm; ARDS: acute respiratory distress syndrome; ARF: acute respiratory failure; bpm: beats per minute; COPD: chronic obstructive pulmonary disease; CPR: cardiopulmonary resuscitation; CRA: cardiorespiratory arrest; CVA: cerebral vascular accident; GI: gastrointestinal; Hb: hemoglobin; HF: heart failure; HR: heart rate; ICU: intensive care unit; LOS: length of stay; Mb: myoglobin; MBP: mean blood pressure; MEP: maximum expiratory pressure; MI: myocardial infarct; MIP: maximum inspiratory pressure; MV: mechanical ventilation; neo: neoplasm; Post-op: postoperative; RR: respiratory rate; RSBI: rapid shallow breathing index; SAPS: simplified acute physiology score; SBP: systolic blood pressure; SD: standard deviation; SIMV: synchronized intermittent mandatory ventilation; T: temperature; TBI: traumatic brain injury; TCT: tracheostomy; Vt: tidal volume; y: years. REFERENCES 1. Condessa RL, Brauner JS, Saul AL, Baptista M, Silva AC, Vieira SR. Inspiratory muscle training did not accelerate weaning from mechanical ventilation but did improve tidal volume and maximal respiratory pressures: a randomised trial. J Physiother. 2013;59(2):101-7. 2. da Silva Guimarães B, de Souza LC, Cordeiro HF, Regis TL, Leite CA, Puga FP, et al. Inspiratory Muscle Training With an Electronic Resistive Loading Device Improves Prolonged Weaning Outcomes in a Randomized Controlled Trial. Crit Care Med. 2021;49(4):589-97. 3. Martin AD, Smith BK, Davenport PD, Harman E, Gonzalez-Rothi RJ, Baz M, et al. Inspiratory muscle strength training improves weaning outcome in failure to wean patients: a randomized trial. Crit Care. 2011;15(2):R84. 4. Pascotini FD, Denardi C, Nunes GO, Trvisan ME, Antunes VD. Treinamento muscular respiratório em pacientes em desmame da ventilação mecânica. ABCS Health Sci. 2014;39(1):12-6. 5. Melo PF, Da Silva V, Vieira L, Lima L, Lira A, Silva PE, et al. High Intensity Inspiratory Muscle Training in Patients with Traumatic Brain Injury Under Mechanical Ventilation: Preliminary Results of a Randomized Controlled Trial. In: A104 Critical Care: improving ICU exercise, rehabilitation, recovery, and survivorship. American Thoracic Society; 2017 May 19-24. Washington, DC. p. A2749-A2749. (American Thoracic Society International Conference Abstracts). [cited 2024 Nov 11]. Available from: https://www.atsjournals.org/doi/epdf/10.1164/ajrccm-conference.2017.195.1_MeetingAbstracts.A2749?role=tab 6. Van Hollebeke M, Poddighe D, Clerckx B, Muller J, Hermans G, Gosselink R, et al. High-Intensity Inspiratory Muscle Training Improves Scalene and Sternocleidomastoid Muscle Oxygenation Parameters in Patients With Weaning Difficulties: A Randomized Controlled Trial. Front Physiol. 2022;13:786575. 7. Roceto Ratti LD, Marques Tonella R, Castilho de Figueir do L, Bredda Saad IA, Eiras Falcão AL, Martins de Oliveira PP. Inspiratory Muscle Training Strategies in Tracheostomized Critically Ill Individuals. Respir Care. 2022;67(8):939-48. **Table 8S:** Summary of group characteristics

Author (year) Country	Cannula type	Method/device used	Characteristics of IMT and comparators
Condessa et al. (2013)^(1)^ Brazil	- Experimental: ETT - Control: ETT	- Experimental: Threshold IMT^a^ - Control: IMT was not performed	Initial training load Experimental: 40% of MIP; Con: NA Training series Experimental: 5 sets of 10 breaths; Con: NA Time of training Experimental: 2x/day, 7x/week; Con: NA
da Silva Guimarães et al. (2021)^(2)^ Brazil	- Experimental: TCT cannula - Control: TCT cannula	- Experimental: POWERbreathe^®^ K-5^b^ electronic inspiratory training device - Control: ‘T-piece’	Initial training load Experimental: 40% of MIP, progressively adjusted until the target load was reached + protocol of progressively lengthening ‘T-piece’ trials, daily re-evaluated; Con: protocol of progressively lengthening ‘T-piece’ trials, daily re-evaluated Training series Experimental: 2 sets of 30 breaths, 2-3 min of rest between sets. Each set consisted of 3 subsets of 10 breaths each. In each subset of 10 breaths, the load started at half of the target, and the last 5 breaths of each subset were run under the target load; Con: NR Time of training Experimental: 1x/day per 7 days; Con: 1x/day. At the end of each training session, patients returned to PSV mode
Martin et al. (2011)^(3)^ United States	- Experimental: TCT tube - Control: TCT tube	- Experimental: threshold PEP^a^ - Control: resistive inspiratory muscle training device Pflex^a^	Initial training load Experimental: threshold inspiratory pressure load between 4-20 cmH_2_O. The training load was set to the highest pressure setting that the subject could consistently open during inspiration through the exaltation port, and progressed daily as tolerated; Con: Pflex device (with 3 mm hole drilled into the device body), which further reduced the pressure required to generate airflow Training series Experimental: 4 sets of 6-10 training breaths per day, with 2-min of rest on MV support between each set; Con: 4 sets of 6-10 breaths, with 2-min of rest on MV support between sets Time of training Experimental: 5x/week; Con: 5x/week
Pascotini et al. (2014)^(4)^ Brazil	- Experimental: TCT tube - Control: TCT tube	- Experimental: Threshold IMT^a^ - Control: IMT was not performed	Initial training load Experimental: 20% of MIP; Con: NA Training series Experimental: 3 sets of 10 breaths, with 2-min of rest between sets; Con: NA Time of training Experimental: 1x/day per 7 days; Con: NA
Melo et al. (2017)^(5)^ Brazil	- Experimental: NR - Control: NR	- Experimental: POWERbreathe^®^ K5^b^ series - Control: IMT was not performed	Initial training load Experimental: 50% of MIP; Con: NA Series of training Experimental: NR; Con: NA Time of training Experimental: NR; Con: NA
Van Hollebeke et al. (2022)^(6)^ Belgium	- Experimental: ETT and TCT - Control: ETT and TCT	- Experimental = POWERbreathe^®^ KH2^c^ - Control = POWERbreathe^®^ KH2^c^	Initial training load Experimental: 30-50% of MIP, adjusted daily to the highest tolerable load; Con: maximum of 10% of MIP with no adjustments to this load during the entire training period Training series Experimental: 4 sets of 6-8 breaths, with a 2-min of rest between sets; Con: 4 sets of 6-10 breaths. Time of training Experimental: 28 days or until weaned from MV; Con: 28 days or until weaned from MV
Roceto Ratti et al. (2022)^(7)^ Brazil	- Experimental 1: TCT tube - Experimental 2: TCT tube - Control: TCT tube	- Experimental 1 and Exp 2: POWERbreathe^®^ KH2^b^ connected to a notebook^d^ equipped with the BreatheLink software (IMT Technologies) - Control: ‘T-piece’	Initial training load Experimental 1: automatically adjusted according to the maximal effort exerted by patients during the first 2 breaths of each training session; Exp 2: 30% of MIP, with daily increments of 10%; Con: NA Training series Experimental 1 and Exp 2: 3 series of 10 breaths guided by RP with 1-min of rest between sets; Con: ‘T-piece’ duration was progressively increased if patients presented no signs of respiratory discomfort, RR ≤30 breaths/min, SaO_2_ ≥ 90%, MBP ≥80mmHg or ≤110mmHg, and HR ≥60 bpm or ≤120 bpm Time of training Experimental 1 and Exp 2: 2x/day, 7x/week, until weaning from MV (liberation from MV for 48 h); Con: NR BIPAP: bilevel positive airway pressure ; CPAP: continuous positive airway pressure; ETT: endotracheal tube; IMT: inspiratory muscle training; Con: Control; MBP: mean blood pressure; MIP: maximum inspiratory pressure; MV: mechanical ventilation; NA: not applicable; NAVA: neurally adjusted ventilatory assist; NR: not reported; Obs: observation; PEP: positive expiratory pressure; PSV: pressure support ventilation; rep: repetitions; RR: respiratory rate; RP: respiratory physiotherapist; SaO_2_: arterial oxygen saturation; TCT: tracheostomy. REFERENCES 1. Condessa RL, Brauner JS, Saul AL, Baptista M, Silva AC, Vieira SR. Inspiratory muscle training did not accelerate weaning from mechanical ventilation but did improve tidal volume and maximal respiratory pressures: a randomised trial. J Physiother. 2013;59(2):101-7. 2. da Silva Guimarães B, de Souza LC, Cordeiro HF, Regis TL, Leite CA, Puga FP, et al. Inspiratory Muscle Training With an Electronic Resistive Loading Device Improves Prolonged Weaning Outcomes in a Randomized Controlled Trial. Crit Care Med. 2021;49(4):589-97. 3. Martin AD, Smith BK, Davenport PD, Harman E, Gonzalez-Rothi RJ, Baz M, et al. Inspiratory muscle strength training improves weaning outcome in failure to wean patients: a randomized trial. Crit Care. 2011;15(2):R84. 4. Pascotini FD, Denardi C, Nunes GO, Trvisan ME, Antunes VD. Treinamento muscular respiratório em pacientes em desmame da ventilação mecânica. ABCS Health Sci. 2014;39(1):12-6. 5. Melo PF, Da Silva V, Vieira L, Lima L, Lira A, Silva PE, et al. High Intensity Inspiratory Muscle Training in Patients with Traumatic Brain Injury Under Mechanical Ventilation: Preliminary Results of a Randomized Controlled Trial. In: A104 Critical Care: improving ICU exercise, rehabilitation, recovery, and survivorship. American Thoracic Society; 2017 May 19-24. Washington, DC. p. A2749-A2749. (American Thoracic Society International Conference Abstracts). [cited 2024 Nov 11]. Available from: https://www.atsjournals.org/doi/epdf/10.1164/ajrccm-conference.2017.195.1_MeetingAbstracts.A2749?role=tab 6. Van Hollebeke M, Poddighe D, Clerckx B, Muller J, Hermans G, Gosselink R, et al. High-Intensity Inspiratory Muscle Training Improves Scalene and Sternocleidomastoid Muscle Oxygenation Parameters in Patients With Weaning Difficulties: A Randomized Controlled Trial. Front Physiol. 2022;13:786575. 7. Roceto Ratti LD, Marques Tonella R, Castilho de Figueir do L, Bredda Saad IA, Eiras Falcão AL, Martins de Oliveira PP. Inspiratory Muscle Training Strategies in Tracheostomized Critically Ill Individuals. Respir Care. 2022;67(8):939-48. **Table 9S:** Summary of findings

Inspiratory muscle training compared to usual care for CCIPs											
Certainty assessment							Summary of findings				
Participants (studies) Follow-up	Risk of bias	Inconsistency	Indirectness	Imprecision	Publication bias	Overall certainty of evidence	Study event rates (%)		Relative effect [95%CI]	Anticipated absolute effects	
							With usual care	With IMT		Risk with usual care	Risk difference with IMT
Inspiratory muscle strength											
252 (4 RCTs)	Serious^a^	Serious^b^	Not serious	Serious^c^	None	⨁◯◯◯ Very low	143	109	-	The mean inspiratory muscle strength was -15.93 to -58 cmH_2_0	MD 10.41 cmH_2_0 lower (19.48 lower to 1.34 lower)
Duration of mechanical ventilation (mean follow-up of 7 days)											
166 (3 RCTs)	Not serious^d^	Not serious	Not serious	Extremely serious^c^	None	⨁◯◯◯ Very low	One study^(1)^ demonstrated a slight difference in MV duration when comparing IMT and usual care. One study^(2)^ reported a decrease in MV duration, and one study^(3)^ found no difference				
Number of severe adverse events and non-serious adverse events (follow-up ranged from 7 to 14 weeks)											
77 (1 RCT)	Not serious	Not serious	Not serious	Extremely serious^c^	None	⨁◯◯◯ Very low	One study^(1)^ considered only hemodynamic changes and had no reported adverse events in both groups				

CI: confidence interval; IMT: inspiratory muscle training; MD: mean difference; MV: mechanical ventilation; RCT: randomized controlled trial.

^a^High risk of bias.

^b^Wide confidence intervals and moderate heterogeneity.

^c^Few included participants.

^d^Due to concerns about the methodological quality. **Table:**

Inspiratory muscle training compared to sham for CCIPs											
Certainty assessment							Summary of findings				
Participants (studies) Follow-up	Risk of bias	Inconsistency	Indirectness	Imprecision	Publication bias	Overall certainty of evidence	Study event rates (%)		Relative effect [95%CI]	Anticipated absolute effects	
							With usual care	With IMT		Risk with usual care	Risk difference with IMT
Inspiratory muscle strength											
110 (2 RCTs)	Not serious	Serious^a^	Not serious	Very serious^b^	None	⨁◯◯◯ Very low	53	57	-	The mean inspiratory muscle strength ranged from -48 to -45.1 cmH_2_0	MD 4.26 cmH_2_0 lower (14.05 lower to 5.53 higher)
Number of severe adverse events and non-serious adverse events (follow-up ranged from 7 to 14 weeks)											
69 (1 RCT)	Not serious	Not serious	Not serious	Extremely serious^b^	None	⨁◯◯◯ Very low	One study^(4)^ reported no evidence of adverse events in both groups				

CI: confidence interval; IMT: inspiratory muscle training; MD: mean difference; RCT: randomized controlled trial.

^a^Wide confidence intervals.

^b^Few included participants. REFERENCES 1. Shosholcheva M, Jankulovski N, Kartalov A, Kuzmanovska B. Abstract PR127: Early Physical Rehabilitation Improves Outcome At Mechanical Ventilated Patients. Anesth Anal. 2016;23(3S):168. 2. Shrestha BK, Qutob HF, Berry M, Files DC, Dhar S, Bowton D, et al. Feasibility and safety of inspiratory muscle training in critically ill intubated patients. Am J Respir Crit Care Med. 2014;189:A3882. 3. Pascotini FD, Denardi C, Nunes GO, Trvisan ME, Antunes VD. Treinamento muscular respiratório em pacientes em desmame da ventilação mecânica. ABCS Health Sci. 2014;39(1):12-6. 4. Martin AD, Smith BK, Davenport PD, Harman E, Gonzalez-Rothi RJ, Baz M, et al. Inspiratory muscle strength training improves weaning outcome in failure to wean patients: a randomized trial. Crit Care. 2011;15(2):R84.
